# High prevalence of functional dyspepsia in nonalcoholic fatty liver disease: a cross-sectional study

**DOI:** 10.1590/1516-3180.2021.0015.R1.14062021

**Published:** 2022-01-17

**Authors:** Érika Cristina Lima, Maria do Carmo Friche Passos, Silvia Marinho Ferolla, Raissa Soares Neves da Costa, Quelson Coelho Lisboa, Lucas Ismael Dias Pereira, Mateus Jorge Nardelli, Vitor Nunes Arantes, Teresa Cristina de Abreu Ferrari, Claudia Alves Couto

**Affiliations:** I MSc. Nurse, Postgraduate Program in Sciences Applied to Adult Health Care, School of Medicine, Universidade Federal de Minas Gerais (UFMG), Belo Horizonte (MG), Brazil.; II MD, PhD. Professor, Alfa Institute of Gastroenterology, Hospital das Clínicas, Universidade Federal de Minas Gerais (UFMG), Belo Horizonte (MG), Brazil.; III MD, PhD. Professor, Postgraduate Program in Sciences Applied to Adult Health Care, School of Medicine, Universidade Federal de Minas Gerais (UFMG), Belo Horizonte (MG), Brazil.; IV MD. Collaborator, Department of Internal Medicine, School of Medicine, Universidade Federal de Minas Gerais (UFMG), Belo Horizonte (MG), Brazil.; V MD, MSc. Collaborator, Department of Internal Medicine, School of Medicine, Universidade Federal de Minas Gerais (UFMG), Belo Horizonte (MG), Brazil.; VI MD. Collaborator, Department of Internal Medicine, School of Medicine, Universidade Federal de Minas Gerais (UFMG), Belo Horizonte (MG), Brazil.; VII MD. Postgraduate Student, Postgraduate Program in Sciences Applied to Adult Health Care, School of Medicine, Universidade Federal de Minas Gerais (UFMG), Belo Horizonte (MG), Brazil.; VIII MD, PhD. Professor, Alfa Institute of Gastroenterology, Hospital das Clínicas, Universidade Federal de Minas Gerais (UFMG), Belo Horizonte (MG), Brazil.; IX MD, PhD. Professor, Alfa Institute of Gastroenterology, Hospital das Clínicas, Universidade Federal de Minas Gerais (UFMG), Belo Horizonte (MG), Brazil.; X MD, PhD. Professor, Alfa Institute of Gastroenterology, Hospital das Clínicas, Universidade Federal de Minas Gerais (UFMG), Belo Horizonte (MG), Brazil.

**Keywords:** Gastrointestinal diseases, Nonalcoholic fatty liver disease, Dyspepsia, Obesity, Diabetes mellitus, Functional gastrointestinal disorders, NAFLD, sIndigestion, Body weight

## Abstract

**BACKGROUND:**

Gastrointestinal (GI) symptoms are frequent complaints from individuals with nonalcoholic fatty liver disease (NAFLD). Dyspepsia is a universal clinical symptom and is among the most common GI complaints observed in the general population, but its prevalence in the population with NAFLD has not been previously investigated.

**OBJECTIVE:**

To compare the prevalence of functional dyspepsia (FD) between patients with NAFLD and controls without liver disease.

**DESIGN AND SETTING:**

Cross-sectional study at the Outpatient Liver Clinic, University Hospital, Belo Horizonte, Brazil.

**METHODS:**

We included 96 NAFLD patients and 105 controls without liver disease. All participants were assessed for GI symptoms in accordance with the Rome III criteria. Evaluation methods included a questionnaire for FD (validated in Brazil), laboratory tests and upper GI endoscopy.

**RESULTS:**

Mean age and sex were similar between the groups. The NAFLD group presented higher frequency of proton-pump inhibitor usage (31.3% vs 4.8%; P < 0.001) and prevalence of FD (25.0% versus 12.4%; P = 0.021). The symptom frequencies were as follows: postprandial distress, 22.9% versus 11.4% (P = 0.030); postprandial fullness, 18.8% versus 10.5% (P = 0.095); early satiation, 8.3% versus 5.7% (P = 0.466); and epigastric pain or burning, 18.8% versus 5.7% (P = 0.004), in NAFLD patients and controls, respectively. Multivariate analysis demonstrated that female sex (odds ratio, OR 6.97; 95% confidence interval, CI: 1.51-32.12; P = 0.013) and NAFLD diagnosis (OR 2.45; 95% CI: 1.14-5.27; P = 0.021) were independently associated with FD occurrence.

**CONCLUSION:**

FD occurs more frequently in individuals with NAFLD than in controls without hepatic disease.

## INTRODUCTION

Nonalcoholic fatty liver disease (NAFLD) is currently considered to be a public health problem in many countries, affecting both adults and children. This condition is characterized by hepatic steatosis, which is detected through ultrasound (US) or histological examination of the liver in individuals without a history of excessive alcohol consumption and with no other causes of liver disease.^1^ NAFLD can progress to nonalcoholic steatohepatitis (NASH), cirrhosis and hepatocarcinoma. Obesity, insulin resistance, type 2 diabetes mellitus (DM) and other components of metabolic syndrome are common related comorbidities.^1^ The global incidence of NAFLD is unknown since it depends on the population studied and on the methods used to diagnose this condition (e.g. liver biopsy, magnetic resonance spectroscopy or US). Despite these limitations, the prevalences of NAFLD and NASH in the general population in Western countries have been estimated to reach 20%-30% and 1%-3%, respectively.^1,2^

NAFLD is considered to be a silent disease with asymptomatic evolution until its advanced stages. Studies have demonstrated a lack of specific symptoms in 45%-100% of patients.^3,4,5^ The diagnosis is made unintentionally in asymptomatic patients through detecting elevated serum aminotransferase levels or steatosis on US performed as a routine test or during investigation of other comorbidities related to NAFLD. However, more recently, it has been suggested that NAFLD patients may present with multiple symptoms related to the gastrointestinal (GI) tract. For example, a high proportion of the patients with NAFLD that was incidentally detected through US examination initially sought medical attention due to the presence of functional GI symptoms.^6^ Moreover, patients with functional dyspepsia (FD) who underwent US have also been described as having high prevalence of fatty liver.^7^ Nevertheless, published data regarding the prevalence of GI symptoms specifically in the NAFLD population are scarce.

Dyspepsia is one of the most frequent GI symptoms observed in the general population. It is defined as a digestive disorder characterized by a set of symptoms related to the upper GI tract, such as pain, burning or discomfort in the upper abdomen, which may be associated with early satiety, postprandial nausea, vomiting, bloating or a feeling of abdominal distention.^8^ The Rome III consensus defines FD as the presence of one or more of the following: epigastric pain or epigastric burning, bothersome postprandial fullness and early satiety with no evidence of a structural disease (including upper endoscopy evaluation) that would explain the symptoms.^9^ Patients with these symptoms but without any structural disease upon diagnostic evaluation probably have FD, even though according to the Rome III guidelines, these criteria should be met during the last three months with symptom onset at least six months before the diagnosis.

## OBJECTIVE

Considering the current increasing burden of NAFLD and the lack of knowledge regarding the characterization of GI symptoms in this population, we conducted this study to test the hypothesis that individuals with NAFLD have higher prevalence of FD than do subjects without fatty liver disease.

## METHODS

### Study population and data collection

This cross-sectional study included 201 subjects who were prospectively selected between August 2015 and December 2016. The patients were consecutively recruited from the Outpatient Liver Clinic, University Hospital, Belo Horizonte, Brazil, after they had been diagnosed with NAFLD. This institution is a referral center within the Brazilian public healthcare system for treating liver diseases. A control group was also formed, and this included 105 individuals without known liver disease. These subjects were the companions of patients treated in the Outpatient Liver Clinic, and they were selected based on their clinical history of no liver diseases. The local ethics committee approved the study (CAAE 26228014.7.0000.5149) on March 12, 2014, and all patients signed an informed consent statement. The sample was obtained according to convenience after inclusion of prospective patients’ inclusion, since the prevalence of FD among NAFLD subjects was unknown.

The diagnosis of NAFLD was established in accordance with the criteria of international guidelines.^10^ The inclusion criteria comprised (a) steatosis on US and/or liver biopsy (performed based on clinical judgment); (b) exclusion of other causes of liver disease (i.e. alcoholic disease, autoimmune disorders, viral hepatitis, hemochromatosis, Wilson’s disease and alpha-1-antitrypsin deficiency); (c) no history of prior gastric or jejunoileal bypass and no exposure to hepatotoxins; (d) no use of steatogenic medications within the past six months; and (e) 18 years of age or older. The inclusion criteria for controls were that they needed to be adults aged between 18 and 75 years and without any history of liver disease. The exclusion criteria for both groups were the presence of a diagnosis of decompensated liver cirrhosis, use of oral contraceptives or nonsteroidal anti-inflammatory drugs, corticosteroid treatment or history of organic GI diseases.

### Clinical and laboratory investigations

Demographic characteristics, anthropometric data, use of proton-pump inhibitors and prevalence of comorbidities were evaluated in all patients. Anthropometric data comprised weight (kg), height (m), waist circumference (cm) (measured midway between the lower limit of the rib cage and the iliac crest, with the participant in a standing position) and body mass index (BMI), which was calculated as weight/height^2^ (kg/m²). For analysis purposes, obesity was defined as BMI ≥ 30 kg/m².

Metabolic syndrome was defined in accordance with the criteria adopted by the International Diabetes Federation:^11^ central obesity (waist circumference ≥ 90 cm in men and ≥ 80 cm in women), along with two or more of the following conditions: hypertriglyceridemia (≥ 150 mg/dl), low high-density lipoprotein (HDL) cholesterol levels (< 40 mg/dl in men and < 50 mg/dl in women), hypertension (systolic blood pressure ≥ 130 mmHg and diastolic ≥ 85 mmHg) and fasting glucose ≥ 100 mg/dl.

All patients who had GI symptoms underwent upper GI endoscopy to investigate the presence of structural disease. We excluded patients with findings suggestive of structural diseases that may cause dyspeptic symptoms, such as peptic ulcers, erosive duodenitis, intestinal metaplasia or gastric mucosal atrophy. Since erosive esophagitis and gastroesophageal reflux disease (GERD) do not usually cause dyspeptic symptoms,^12^ they were not excluded. Nonspecific findings such as enanthematous gastritis/pangastritis and hiatal hernia were described. Esophageal varices were considered to be manifestations of portal hypertension related to progressive steatohepatitis, as long as a diagnosis of NAFLD was previously present.

*Helicobacter pylori* infection was tested by means of histopathological assessment, and individuals with positive tests were treated for its eradication. Patients with persistent dyspeptic symptoms after six months, despite adequate *H. pylori* treatment proved through a respiratory test, were diagnosed as presenting FD.^13^

The laboratory assessment included total cholesterol and fractions, triglycerides, fasting blood glucose and insulin, glycohemoglobin, aspartate aminotransferase (AST), alanine aminotransferase (ALT), gamma glutamyl transferase (GGT), albumin, hemoglobin and platelet count. Insulin resistance in nondiabetic patients was calculated using the homeostatic model assessment index (HOMA), i.e. serum insulin (μU/ml) × fasting glucose (mmol/l)/22.5, and presence of insulin resistance was defined as HOMA values ≥ 3. Presence of DM was diagnosed if patients were on regular oral hypoglycemic drugs and/or insulin, and/or they had a fasting glucose level ≥ 126 mg/dl on two different occasions.

The NAFLD fibrosis score was calculated for all NAFLD patients. This score is a noninvasive method for determining the presence of advanced liver fibrosis in these patients. Scores above 0.676 indicate advanced liver fibrosis, and scores below -1.455 indicate the absence of advanced liver fibrosis.^14^

### Rating of functional dyspepsia between the groups

The presence of GI symptoms was assessed by administering a questionnaire adapted from the criteria proposed in the Rome III^15^ consensus, which has been validated for use in the Portuguese language.^16^ The interpretation was also based on the Rome III definitions for functional disorders. Therefore, the criteria for all diagnosed symptoms had to be met within the last three months and symptom onset needed to be at least six months ago. Functional gastroduodenal disorders such as dyspepsia were diagnosed when there was no evidence of structural disease in upper endoscopy examination, or of abnormal behavior (e.g. self-induced vomiting), central nervous system abnormalities or metabolic diseases that could explain the symptoms.

### Statistical analysis

Statistical analyses were performed using the Statistical Package for the Social Sciences (SPSS) software, version 18 (SPSS Inc, Chicago, Illinois, United States). Categorical variables were presented as frequencies and percentages. Continuous variables were expressed as the mean ± standard deviation when the data were normally distributed, while the median and interquartile range were used for variables with skewed distribution.

For univariate analyses, continuous variables were compared between groups using the nonparametric Mann-Whitney U test. To compare proportions, the chi-square test or Fisher’s exact test was used, as appropriate. For the multivariate analysis, we used logistic regression, with the backward method, to verify predictors of FD and confounding factors. The results were presented as odds ratios (ORs) and 95% confidence intervals (95% CIs). The variables were removed from the model one by one until only variables with a P-value < 0.05 remained. Significance was indicated by a P-value < 0.05.

## RESULTS

### Characteristics of the patients

The present study included 96 patients with NAFLD with a mean age of 55.9 ± 12.7 years and 105 controls with a mean age of 55.2 ± 12.8 years. The demographic characteristics, anthropometric data and prevalence of comorbidities are shown in Table 1.

**Table 1.** Demographic, anthropometric and clinical data on the NAFLD patients and controls

Variable	Groups		P-value
	NAFLD (n = 96)	Control (n = 105)	
Proton-pump inhibitor use	30/96 (31.3)	5/105 (4.8)	< 0.001^†^
Female sex	78 (81.3)	82 (78.1)	0.579^†^
Age (years)	59 (49.5-64.0)	58 (48.5-64.0)	0.638^§^
BMI (kg/m^2^)	32 (28-36)	26 (23-29)	< 0.001^§^
Obesity (BMI ≥ 30)	62/94 (66)	22/104 (21.2)	< 0.001^‡^
Central obesity	93/96 (96.9)	48/105 (45.7)	< 0.001^†^
Hypertension	67/95 (70.5)	36/105 (34.3)	< 0.001^†^
Diabetes	40/95 (42.1)	14/105 (13.3)	< 0.001^†^
Hypertriglyceridemia	49/90 (54.4)	13/91 (14.3)	< 0.001^†^
Low HDL cholesterol	43/88 (48.9)	11/91 (12.1)	< 0.001^†^
Metabolic syndrome	71/95 (74.7)	13/105 (12.4)	< 0.001^†^

Data are expressed as absolute numbers (percentages) and medians (interquartile ranges). NAFLD = nonalcoholic fatty liver disease; BMI = body mass index; HDL = high-density lipoprotein; ^†^chi-square test; ^‡^Fisher’s exact test; ^§^Mann-Whitney U test.

Six patients were not included, based on the following exclusion criteria: four patients had Crohn’s disease (two NAFLD subjects and two controls) and two patients had previously undergone gastroduodenal anastomosis (NAFLD group). Upper gastrointestinal endoscopy revealed peptic ulcers and/or erosive duodenitis in three patients in the NAFLD group. These patients were not included.

There was no difference between the groups regarding sex distribution or median age. NAFLD patients presented significantly higher frequencies of obesity, hypertension, DM, hypercholesterolemia, low HDL-cholesterol levels, metabolic syndrome and use of proton-pump inhibitors than did the controls (Table 1). In the whole sample, 35 patients had been using omeprazole: 30 NAFLD patients (13 with GERD, 10 with FD, seven with both conditions and 14 who were using this drug for other indications) and five control individuals (two had GERD, none had FD and three were using this drug for other indications).

DM was observed in 42.1% of the NAFLD patients. NAFLD fibrosis score analysis demonstrated that, out of the 96 NAFLD patients, 41 (42.7%) did not have significant fibrosis and 13 (13.5%) presented significant fibrosis; in 42 (43.8%) patients, the stage of fibrosis could not be determined from the score.

### Functional gastrointestinal symptoms

All the patients with dyspeptic symptoms underwent upper endoscopy in order to map any presence of organic diseases, as shown in Table 2 (five control individuals did not have this test because they refused to undergo the procedure). Although FD and epigastric burning or pain occurred more frequently in the NAFLD patients, the frequencies of *Helicobacter pylori* infection, gastritis and pangastritis were similar in the two groups.

**Table 2.** Comparison between endoscopic findings among NAFLD patients and controls with dyspeptic symptoms

Variable	Groups		P-value
	NAFLD (n = 27)	Control (n = 9)	
Peptic ulcers	0 (0.0)	0 (0.0)	1.000^†^
Erosive duodenitis	0 (0.0)	0 (0.0)	1.000^†^
Erosive esophagitis	5 (18.5)	0 (0.0)	0.302^†^
Enanthematous gastritis	7 (25.9)	4 (44.4)	0.409^†^
Enanthematous pangastritis	14 (51.9)	1 (11.1)	0.051^†^
Esophageal varices	2 (7.4)	0 (0.0)	1.000^†^
Hiatal hernia	2 (7.4)	1 (11.1)	1.000^†^
*Helicobacter pylori* infection	8 (29.6)	1 (11.1)	0.266^†^

Data are expressed as absolute number (percentage); ^†^Fisher’s exact test.

Out of the 27 NAFLD patients who underwent upper endoscopy, eight were diagnosed with *Helicobacter pylori* (29.6%) and were treated with conventional therapy. After six months of treatment, three of these patients achieved resolution of the dyspeptic symptoms, while the other five had persistent symptoms, despite undergoing a respiratory *Helicobacter pylori* test that confirmed that the treatment had been adequate. Patients with resolution after *Helicobacter pylori* eradication were not considered to have had FD.

**Figure 1.**
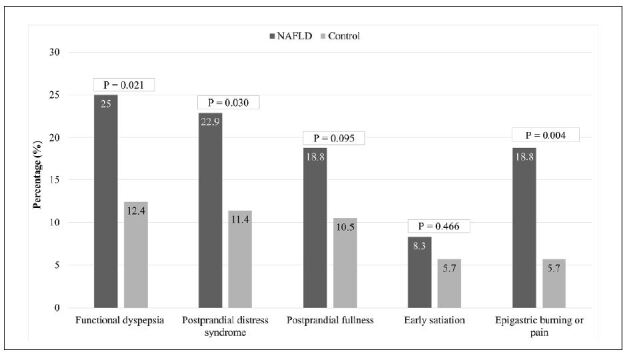
Comparison of frequencies and types of functional dyspepsia syndromes and symptoms between nonalcoholic fatty liver disease (NAFLD) and control groups. Chi-square test.

Figure 1 shows the frequency and type of FD in the NAFLD individuals and controls, respectively: FD, 24 (25.0%) and 13 (12.4%) (P = 0.021); postprandial distress syndrome, 22 (22.9%) and 12 (11.4%) (P = 0.030); postprandial fullness, 18 (18.8%) and 11 (10.5%) (P = 0.095); early satiation, 8 (8.3%) and 6 (5.7%) (P = 0.466); and epigastric burning or pain, 18 (18.8%) and 6 (5.7%) (P = 0.004).

For better characterization of the patients with FD, we compared the individuals with and without FD inside each of the groups (i.e. NAFLD and controls) according to age, sex and features of metabolic syndrome (Table 3). Although the overall NAFLD group presented higher frequency of obese patients (Table 1) than the control group, when the subjects in each group were separated according to the presence or absence of FD, the frequencies of DM, central obesity and other metabolic features were similar among those with NAFLD, and also inside the control group.

**Table 3.** Comparison between patients with and without functional dyspepsia, according to age, sex and features of metabolic syndrome

Variable	NAFLD (n = 96)			Control (n = 105)		
	FD (n = 24)	No FD (n = 72)	P-value	FD (n = 13)	No FD (n = 92)	P-value
Female	23 (95.8)	55 (76.4)	0.037*^†^	12 (92.3)	70 (76.1)	0.289^‡^
Age (years)	56 (42-63)	60 (53-65)	0.140^§^	61 (44-65)	58 (50-64)	0.489^§^
Central obesity	23 (95.8)	70 (97.2)	> 0.999^‡^	6 (46.2)	42 (45.7)	0.973^†^
Hypertension	19 (79.2)	48 (67.6)	0.283^†^	5 (38.5)	31 (32.7)	0.761^‡^
Diabetes	10 (41.7)	30 (42.3)	> 0.999^†^	1 (7.7)	13 (14.1)	> 0.999^‡^
Hypertriglyceridemia	16 (66.7)	33 (45.8)	0.091^†^	0 (0.0)	13 (14.1)	0.354^‡^
Low HDL-c	15 (62.5)	28 (38.9)	0.036*^†^	0 (0.0)	11 (13.8)	0.348^‡^
Metabolic syndrome	20 (83.3)	51 (70.8)	0.262^†^	1 (7.7)	12 (13.0)	> 0.999^‡^

NAFLD = nonalcoholic fatty liver disease; FD = functional dyspepsia; HDL-c = high-density lipoprotein cholesterol. Data are expressed as number (percentage) and median (interquartile range); ^†^chi-square test; ^‡^Fisher’s exact test; ^§^Mann-Whitney U test.

Multivariate analysis was performed in order to investigate predictors for FD occurrence in the whole population studied, by adding the variables of age, sex and NAFLD diagnosis to a logistic regression model. After adjustment, the variables independently associated with FD occurrence were female sex (OR 6.97; 95% CI 1.51-32.12; P = 0.013) and NAFLD diagnosis (OR 2.45; 95% CI 1.14-5.27; P = 0.021).

The NFS categories were not associated with any functional gastrointestinal symptom or disorder: FD (P = 0.689), postprandial distress syndrome (P = 0.784), postprandial fullness (P = 0.944), early satiation (P = 0.612) and epigastric pain/discomfort syndrome (P = 0.489).

## DISCUSSION

In this study, we found high prevalence of FD, according to the Rome III criteria, in the NAFLD group in comparison with its prevalence in the control group without hepatic disease. The frequency of FD was 25.0% in the NAFLD group and only 12.4% in the control group. The prevalence of FD in the control group was similar to what had previously been described in the general population, which ranged from 5.3% to 20.4%.^17^ Although postprandial distress syndrome was more frequent among the patients with FD in the NAFLD group, the frequencies of early satiation and postprandial fullness were similar between the NAFLD and control individuals. The reasons for these findings are unknown and should be addressed in future studies. Corroborating our findings, a recent study that included 195 patients with FD showed high prevalence of associated NAFLD (67%), diagnosed through US.^7^

Our results also showed that a higher percentage of individuals with NAFLD used proton-pump inhibitors and had epigastric burning or pain complaints, than among the controls. To our knowledge, this was the first study evaluating FD according to the Rome III criteria among NAFLD patients. Two previous studies showed higher prevalence of GERD among NAFLD patients,^18,19^ but no study had evaluated functional GI symptoms. We did not investigate functional heartburn because although all the patients with this complaint underwent endoscopy, they were not subjected to further investigations in order to make differential diagnoses regarding this condition. Thus, all the subjects with normal endoscopy results and complaints of heartburn were considered to have GERD. Interestingly, a recent meta-analysis showed high frequency of dyspepsia among subjects with GERD symptoms, which may suggest that that these conditions can overlap.^20^

A heterogeneous group of pathophysiological mechanisms has been implicated in the pathogenesis of FD, including delayed gastric emptying, antral hypomotility, impaired intestinal motility, decreased gastric accommodation, increased visceral sensitivity, abnormal sensitivity to carbohydrates, poor fatty acid duodenal digestion, infiltration of the digestive tract by immune cells and psychological factors. Despite years of intense research, many controversies about the role of these factors and their causal relationship with FD symptoms remain to be elucidated.^20^

Although the pathogenesis of NAFLD has not been fully elucidated, it is well known that this condition is strongly associated with insulin resistance, obesity and dyslipidemia.^1^ Additionally, previous studies demonstrated that FD is associated with central obesity and DM. DM patients frequently report GI symptoms such as postprandial fullness, heartburn, bloating, abdominal pain, early satiety, vomiting and nausea. These symptoms were previously attributed to diabetic gastropathy as an expression of autonomic neuropathy; however, more recent data have suggested that those symptoms are probably due to multifactorial mechanisms.^21,22^ Indeed, controversies regarding the association of DM with GI symptoms still exist. Some studies^23,24^ did not show any differences in the prevalence of GI symptoms between individuals with and without DM, except for lower prevalence of heartburn in individuals with type 1 DM. In contrast, in other investigations,^25,26^ subjects with DM reported significantly more GI symptoms than did control individuals without DM. However, those authors did not use the Rome III criteria for diagnosing FD. We did not find any association between FD and DM within the NAFLD group, or among the controls (Table 3). Female sex has also been associated with FD in diabetic and control populations.^26^

It has been suggested that obesity may cause dyspeptic symptoms by means of different mechanisms, such as alterations in the function of GI neuropeptides;^27^ excess visceral adiposity, which may increase intra-abdominal pressure; and secretion of adipokines and proinflammatory cytokines by visceral adipose tissue.^28^ However, the epidemiological data linking obesity to functional GI disorders are inconsistent.^29^ Although it is well established that obesity is associated with GERD, it remains unclear whether obesity is a risk factor for common functional GI disorders.^30^

Recent studies have demonstrated an association between high BMI and increased risk of FD among females.^31,32^ In the current study, we found an association between female sex and FD, thus corroborating the results from previous studies. Interestingly, one study identified a positive correlation between visceral adiposity and FD. Regarding GI symptoms, only epigastric pain was found to be associated with visceral adiposity.^33^ Those results were different from ours, as our NAFLD patients and controls with FD did not present higher frequency of central obesity, considering each group individually (Table 3).

Our study had limitations that should be noted. Firstly, there was subjectivity in applying the questionnaire and there are inherent clinical difficulties in making assertive diagnoses of functional disturbances. On the other hand, the Rome III questionnaire has been validated in Brazil. Additionally, a specific team was trained for individual and standardized administration of questionnaires. This strength is relevant in comparison with other observational studies in which the questionnaires were administered online or by telephone, or were handed to patients to be returned later.^31,34^ Furthermore, we used the Rome III criteria instead of Rome IV because our team already had experience with its administration; and because Rome IV only presents minor changes in relation to Rome III. These changes were an attempt to increase the specificity of appropriate patient inclusion in clinical trials, whereas in clinical practice this precision may not be required.^35^ Lastly, the controls did not undergo abdominal ultrasonography for diagnosing NAFLD. Thus, the study results may constitute an underestimation, considering that after excluding possible controls with undiagnosed NAFLD, the association between FD and the group with diagnosed fatty liver could even have been stronger than we found. Further studies are needed to confirm this.

## CONCLUSION

In conclusion, the present study provides new evidence regarding the association between FD and NAFLD. The prevalence of FD was higher among individuals with NAFLD. Further studies are required in order to validate these observations and to establish optimal strategies for managing dyspeptic symptoms in these individuals.
